# Assessment of Posterior Segment Using Spectral Domain OCT in Highly Myopic Eyes

**DOI:** 10.2174/1874364101711010334

**Published:** 2017-11-22

**Authors:** Heba Radi AttaAllah, Ismail Ahmed Nagib Omar, Ahmed Shawkat Abdelhalim

**Affiliations:** Ophthalmology Department, Lecturer of Ophthalmology, Minia University Hospital, Faculty of Medicine, Minia University, Al-Minya, Egypt

**Keywords:** Dome shaped macula, Foveoschisis, High myopia, Myopic traction maculopathy, Myopic macular hole, Myopic CNV

## Abstract

**Purpose:**

Spectral Domain Optical Coherence Tomography (SD-OCT) was used to evaluate retinal and vitreo-retinal changes that occur in highly myopic patients.

**Methods:**

This prospective study included 472 eyes of 472 patients suffering from high myopia (> -6.00 D), between May 2012 and December 2015. All patients were examined, using Cirrus HD OCT (Zeiss Cirrus TM HD-OCT model 4000), to detect any retinal or vitreo-retinal interface abnormalities.

All obtained data was analyzed using Statistical Package for the Social Sciences software version 17 (SPSS Inc, Chicago, IL, USA) and the paired two-sided t-test. Bivariate correlations were performed between different parameters using the Spearman correlation coefficient (r).

**Results:**

Mean spherical equivalent (MSE) was -13.11± 4.35D. Mean axial length (AL) was 28.5±1.62 mm. Posterior vitreous detachment (PVD) was the most frequent OCT finding; representing 33.4% of the cases, 13.7% of them were associated with macular traction. A statistically significant positive correlation was found between AL and MTM, full thickness macular hole, PVD with traction, and dome shaped macula (r = 0.49 and P = 0.001, r = 0.422 and P = 0.0001, r = 0.25 and P = 0.03, r=0.475, P=0.001 respectively)

**Conclusion:**

OCT is a valuable tool in detecting retinal and vitreo-retinal interface abnormalities in highly myopic eyes, and it can be used for follow up of those patients to avoid advanced retinal damage.

## 1 INTRODUCTION

High or pathologic myopia is defined as a refractive error of ≥ -6.00 diopters (D) and an axial length >26 mm [1]. In pathologic myopia, fundus changes are numerous and related to the degree of myopia, axial length of the globe, presence of a posterior staphyloma and age [2].

The unique combination of retinal traction generated by the epiretinal membrane (ERM) and/or residual focal vitreomacular traction (VMT), together with the complex and distinctive anatomy of degenerative myopia, leads to the frequent presence of macular damage in these eyes, such as retinoschisis, lamellar holes, macular hole formation and posterior retinal detachment. Such findings may be difficult to diagnose on routine fundus examination or by fluorescein angiography [3]. However, the recent increased use of optical coherence tomography (OCT) has revealed the presence of posterior pole related conditions that are difficult to visualize with other imaging techniques.[2]

While working in Upper Egypt, we have observed an increase in the number of highly myopic patients; a considerable number of those patients prefer to have their refractive errors corrected by refractive surgeries, rather than wearing eye glasses, in pre-operative evaluation of those patients we noted significant visual impairment, in some patients, not explained by routine fundus examination. In this study, we aimed to evaluate retinal and vitreo-retinal changes using spectral domain (SD) OCT in such highly myopic eyes with recent visual complaints, either referred for refractive surgery preparation or attending our outpatient clinic. Furthermore, we aimed to study any abnormalities that might be missed on routine examination and that could lead to significant retinal damage, thus keeping such patients on regular follow-up visits, with the aim of decreasing the number of visually disabled, highly myopic patients.

## 2 SUBJECTS AND METHODS

This prospective study was done between May 2012 and December 2015. It included 472 eyes of 472 patients who attended the ophthalmic outpatient clinic in Minia University hospital, and Minia investigation eye center; 40.4% (191) were male and 59.5% (281) were female. Age ranged between 22 and 65 years, with a mean and standard deviation of 50.38 ± 11.4.

We included all symptomatic patients with high myopia (> -6.00 D), who had recent metamorphopsia or unexplained visual symptoms on routine examination, only one eye for each patient was selected for analysis (the eye with more findings, or with recent complaint). Patients with media opacities that may have interfered with scan signal strength, and with a previous history of intraocular surgeries, or with other ocular or systemic diseases that could affect the macular area (dystrophy, age related macular degeneration, diabetic retinopathy, etc.) were excluded from this study.

Patients' assessment included: Best corrected visual acuity, slit lamp examination for anterior segment evaluation, fundus examination using +78 D lens, a cycloplejc refraction using the Nidek autoref/keratometer (LS 900, Haag-Streit Diagnostics, Switzerland), and fluorescein angiography using IMAGE net 2000™; fundus camera (TOPCON Corporation, Japan). Axial length measurement was made using al Quantel medical Aviso (A&B scan Ophthalmic Echography, 2008, France).

OCT examination, using Cirrus HD OCT (Zeiss Cirrus TM HD-OCT model 4000), was performed after appropriate pupillary dilation using cyclopentolate 1% eye drops; high definition HD 5line Raster scan was selected and used at four different orientations: oblique (45°and 135°), vertical scans (90°) and horizontal scans (180°), to scan the posterior pole thoroughly (at the macular area, and nasally). The scan length was 9 mm to cover a larger examination area (except for the vertical scan, where we used a 6 mm scan). All OCT examinations were performed by one observer (H.R.). OCT imaging was optimized by maintaining a good tear film, by applying artificial tears.

This study gained approval from the Local Research Ethics Committee and it was in accordance with the Helsinki Declaration. All patients gave their written consent.

## 3 STATISTICAL ANALYSIS

All obtained data was analyzed using Statistical Package for the Social Sciences software version 17 (SPSS Inc, Chicago, IL, USA) and the paired two-sided t-test. A P-value < 0.05 was considered to be statistically significant. Bivariate correlations were performed between different parameters using the Spearman correlation coefficient (r).

## 4 RESULTS

This study included 472 eyes of 472 patients. Patients’ age, spherical equivalent (SE), and axial length (AL) are shown in Table **1**.

Abnormal macular findings were observed by two observers, recorded and analyzed.

**Fig. (1):**
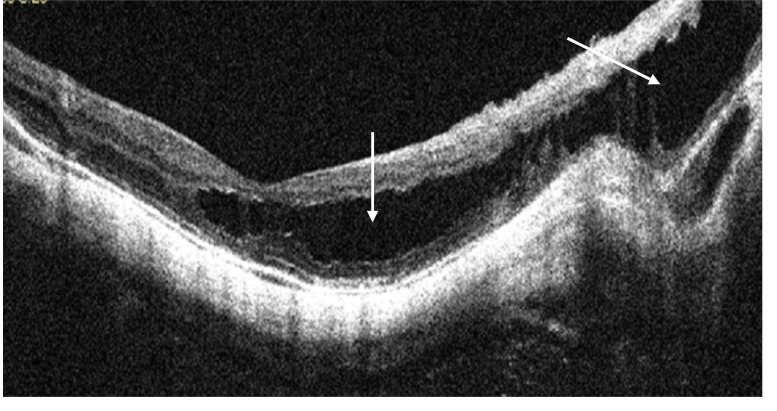
HD OCT scan showing myopic foveoschisis, (white arrows).

Myopic foveoschisis (MF) was defined as a separation of intraretinal layers, predominantly outer layers, into a thinner outer layer and a thicker inner layer, with hyper-reflective bridging columns, and subsequent retinal destruction [4] (Fig. **1**).

**Fig. (2):**
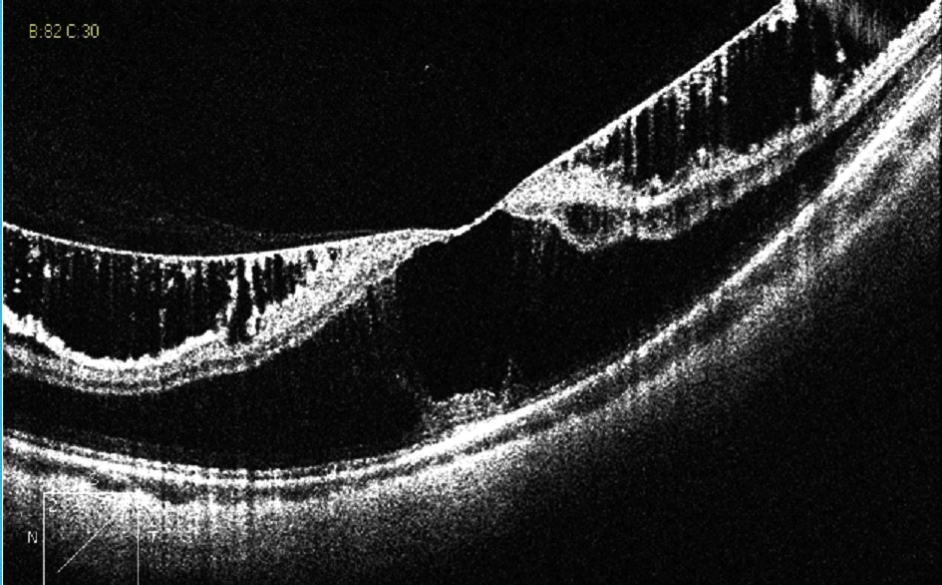
HD OCT scan showing myopic traction maculopathy (MTM).

Myopic traction maculopathy (MTM; Fig. **2**), was diagnosed as follows: the presence of an epiretinal membrane, vitreomacular traction due to incomplete vitreomacular separation, retinal thickening with or without cystoid edema, separation of the neurosensory retina into two or more layers, retinal detachment, lamellar or full-thickness macular holes [2, 5].

A paravascular retinal cyst appears as a small hollow space mainly around large retinal vessels [4], as seen in Fig. (**3**).

Retinal traction was subdivided into: an epiretinal membrane with multifocal attachments causing tangential traction, and incomplete vitreomacular separation (partial posterior vitreous detachment) causing anteroposterior vitreomacular traction [4].

**Fig. (4):**
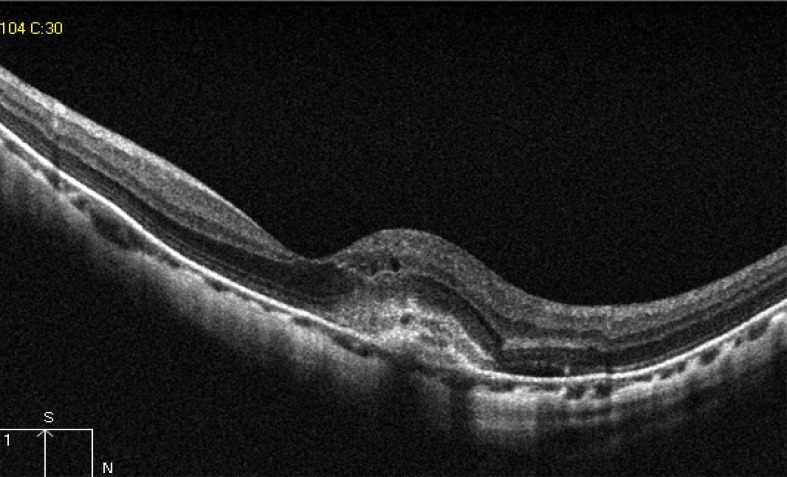
HD OCT scan showing active myopic CNV, which was seen in (18.0%).

Active CNV (choroidal neovascularization) was diagnosed as a hyper-reflective lesion above the RPE (type 2 CNV), with fuzzy borders, disrupted or altered’ IS/OS junction, with minimal subretinal or intraretinal edema fluid (Fig. **4**). In the fibrotic stage, only the surface of the CNV shows high reflectivity, with marked attenuation below the surface [6].

**Fig. (6):**
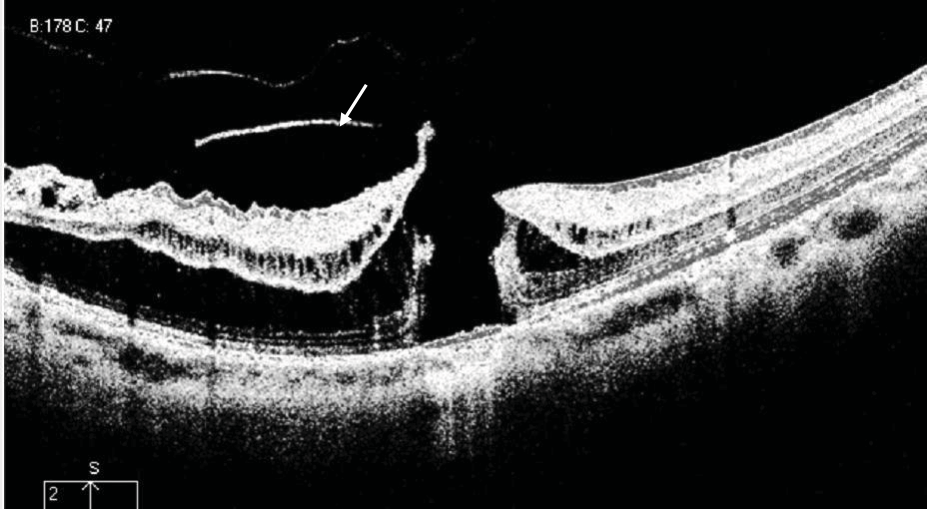
HD OCT scan showing full thickness macular hole (FTMH), which was seen in (8.2%), in this case there is an evidence of antero-posterior vitreo-retinal traction at the inferior retinal edge (white arrow).

**Fig. (7):**
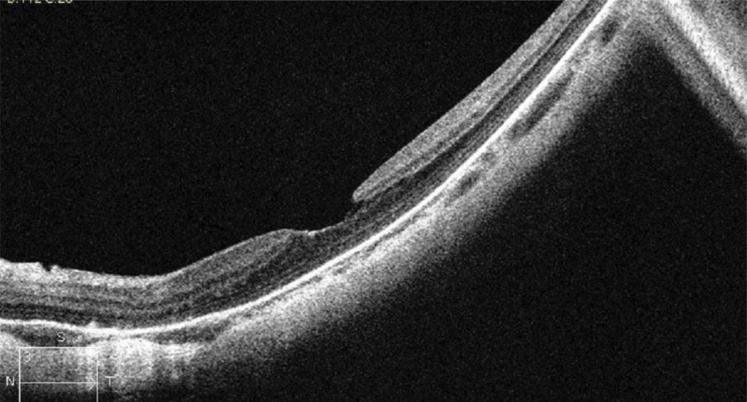
HD OCT scan showing lamellar macular hole that was seen in (4.6%).

**Fig. (8):**
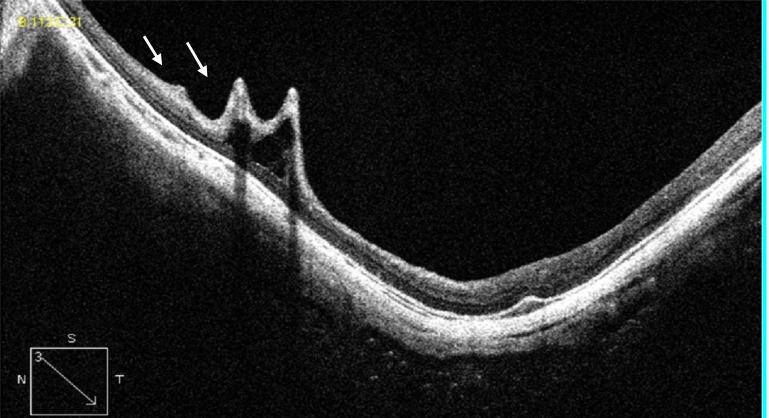
HD OCT scan showing 2 vascular microfolds (white arrows).

Tractional internal limiting membrane (ILM) detachment was defined as a hyper-reflective membrane associated with Muller's cell columns bridging from it to the rest of the retinal layers [7], seen in Fig. (**5**). A full-thickness macular hole (Fig. **6**), lamellar macular hole (Fig. **7**), and vascular microfolds (Fig. **8**) are shown in the indicated figures.

Dome shaped macula was defined on vertical OCT scans, as inward bulge of RPE of more than 50 µm, above a presumed tangential line joining the outer border of RPE, at the bottom of the posterior staphyloma [8], as seen in Fig. (**9**).

The frequency of OCT findings is shown in Table **2**. On further analysis, it was found that 287 eyes (60.8%) had an axial length >28 mm, while 90 eyes (19%) had an axial length >30 mm, the frequency of some OCT findings as regard to the AL is shown in Table **3**.

A fair positive correlation was found between AL and MTM (r = 0.49 and P = 0.001), a fair positive correlation was found between AL and full thickness macular hole (r = 0.422 and P = 0.0001), a fair positive correlation was found between AL and PVD with traction (r = 0.25 and P = 0.03), also a fair positive correlation was found between AL and dome shaped macula (r=0.475, P=0.001). [Grades for correlation (r ): 0.00–0.24 (weak or no association),0.25–0.49 (fair association), 0.50–0.75 (moderate association), and > 0.75 (strong association)],A P value less than or equal to 0.05 was considered significant.

## 5 DISCUSSION

Pathologic myopia has a high disease burden and has been found to be the first, second or third most frequent cause of blindness in several population-based studies [9].

In this prospective study, a sample of 472 eyes of 472 consecutive patients with high myopia on routine examination with recent visual impairment was analysed. Using high resolution OCT, we looked for retinal and vireo-retinal interface changes, where we focused on the changes that endanger the visual function of the patient and are associated with retinal damage.

One of these changes is MTM, which was found in 61 eyes (12.9%). As regard to the AL, 48 eyes with MTM had an axial length >28 mm, and 45 eyes with MTM had an axial length >30 mm, which means that nearly 50% of eyes with an axial length >30 mm had MTM.

Prior to optical coherence tomography, MTM was difficult to distinguish from shallow retinal detachments and macular holes because slit-lamp biomicroscopy is limited in patients with myopic chorioretinal atrophy [10].

In previous studies of the natural course of the disease; some eyes may remain stable for many years, while others progress to more serious complications such as full-thickness macular holes or foveal detachments [11-13].

Spontaneous resolution of MTM has also been reported following posterior vitreous detachment. Risk factors for worse prognosis and progression to serious complications include severity of macular retinoschisis [14], and the presence of premacular structures such as epiretinal membranes [15].

While some researchers postulate that vitreous traction has major contribution in MTM pathogenesis [16, 17], believe that the extension of the eye axis and the formation of staphyloma may also be major contributing factors [18]. Our results agree with the second opinion as there was a statistically significant positive correlation between AL and MTM (r = 0.49 and P = 0.001).

Early MTM can be divided into two types: affecting vision and unaffecting vision. Patients with affected vision may seek medical attention earlier than those with unaffected vision, thus increasing their chances of preventing the disease from reaching a more advanced stage [19, 20].

Some authors advocate that surgical treatment is indicated in MTM when VA is impaired or the patient complains of visual disturbances [15]. Another study shows persistent photoreceptor defects and irregular choroidal detachments in MTM patients with poor post-operative visual outcomes, and recommends the timing of surgery for when there is threatened disruption of the outer retina on OCT [21].

The effect of epiretinal traction on highly myopic eyes is more serious than on non-myopic eyes, because it produces more pronounced retinal damage. Therefore, it should be taken as a major risk factor for retinal damage in those patients [22]. In this study, 139 eyes (29.4%) had retinal traction, ERM with tangential traction was found in 74 eyes (15.6%), and partial posterior vitreous detachment with antero-posterior traction was found in 65 eyes (13.7%). A statistically significant positive correlation was found between AL and PVD with traction (r = 0.25 and P = 0.03).

Gomaa and Abo Hussein, who conducted their study on 100 highly myopic Egyptian patients, report that epiretinal membranes were present in 65 eyes (they did not mention whether all epiretinal membranes were associated with tangential traction or not), while vitreomacular traction (anterior-posterior traction) was detected in ten eyes [23].

The importance of OCT was to detect subtle degrees of traction that could not be noticed on routine biomicroscopic examination.

As demonstrated by Gallemore *et al*., retinal damage is defined by the presence of retinoschisis, lamellar or full-thickness macular hole and shallow retinal detachment [24]. Accordingly, in the current study, we found retinal damage in 34.9% of cases (165 eyes; lamellar macular holes in 22 eyes, full-thickness macular holes in 39 eyes and foveoschisis in 104 eyes). Such a high frequency of retinal damage in a myopic population corresponds to the 34% reported by Takano and Kishi in a smaller sample (32 eyes) [25].

In this study, myopic foveoschisis was found in 104 eyes, representing 22% of the cases, 100 eyes had an axial length >28 mm, alone or associated with other lesions. It is characterized by an intraretinal cleavage in a myopic posterior staphyloma, and it can only be detected by OCT.

Smiddy suggested that myopic maculoschisis, foveal schisis and vitreoschisis in high myopia should all fall under the family of MTM [26].

Gaucher *et al*. report that the risk of worsening vision seems to increase when MF is associated with premacular structures such as epiretinal membranes or a partially detached vitreous cortex [15].

Shimada *et al*. found that half of patients with MF have been reported to develop retinal detachment or macular holes within two or more years of follow-up, and recommend that serial OCT examinations should be performed in these patients [11]. Unfortunately, in our study we couldn’t obtain such data, as we were concerned with the frequency of findings, more than follow up of these findings.

Shimada *et al*. suggest in another longitudinal study of five eyes with retinoschisis that progressed to a foveal retinal detachment using time-domain OCT that inward traction was transmitted to the outer retina through the foveal columnar structures in the retinoschisis layer [27].

Tractional ILM detachment was reported in 50 eyes (10.5%) in the current study. Sayanagi *et al* [28], report that this phenomenon may be an important contributor to the separation of the inner layers of the neural retina resulting in macular retinoschisis. It also seems that traction myopic maculopathy, as described by Panozzo and Mercanti in 2004, is a variation of longstanding tractional ILM detachment, with loss of column bridges due to extreme traction and retinal atrophy [2].

The cause of the ILM detachment is still uncertain. In Goma and Abo Hussein’s study, they record ILM detachments in seven out of 100 eyes; they were always in the peripheral macula (extra foveal), where there were retinal vessels including arterioles [23], but in this series some cases (13 eyes) had ILM detachments reaching juxta and parafoveal locations starting from peripheral locations.

Axial length elongation and/or formation of posterior staphyloma in highly myopic eyes may generate the inward traction force exerted by these factors. This is strongly supported by the remission of the retinoschisis after vitrectomy combined with ILM peeling, which theoretically releases the traction forces exerted by the posterior vitreous cortex and the ILM and partly by the retinal vessels in the area from which the ILM was peeled [29].

Macular holes were found in 61 eyes (12.8%); 39 eyes had full-thickness macular holes, 27 eyes had an axial length >28 mm, while 23 eyes had an axial length >30 mm. There was a statistically significant positive correlation between AL and full thickness macular holes (r = 0.422 and P = 0.0001).

Prognosis is generally better in cases involving only high-myopia macular holes without foveoschisis than in cases with both [30].

Retinal detachment (shallow) was found in 3.8% (18 eyes) of the cases. Almost all of them (16 eyes) had an axial length >28 mm. In 2004, Panozzo and Mercanti reported 1.6% of shallow RD in a sample of 125 myopic eyes [2], but in the Baba *et al*. study they found 9% of 134 eyes [31].

In our study, 119 eyes (25.2%) had choroidal nonvascular membranes, 34 eyes (7.2%) were in an inactive form and the remaining were active. Only 39 eyes of those with active CNV had an axial length >28 mm, and 32 eyes had an axial length of >30 mm.

Ikuno *et al* [32] made a study of 23 consecutive patients with bilaterally high myopia (axial length ≥26.5 mm, or refractive error ≤8 D) and they hypothesized that axial length elongation alone did not cause myopic CNV, and that there may be other latent factors. They confirmed that the axial length and refractive error did not differ between the affected eyes and fellow eyes, indicating that these factors were not major risk factors of myopic CNV. They also found that in eyes with myopic CNV the sub foveal and inferior choroid were significantly thinner than in fellow eyes; furthermore, they found that the posterior staphyloma was steeper in eyes with myopic CNV, explaining the greater choroidal thinning caused by more mechanical stress related to stretching at the macula [32].

Dome shaped macula, first described by Gaucher *et al*. as inward bulge of the macula within the concavity of a posterior staphyloma in highly myopic eyes based on OCT observations [33]. In the current study, it was found in 58 eyes (12.2%). Its prevalence is estimated to be 10.7% by an European study and 9.3% by a Japanese study [4]. Development of dome shaped macula can be explained by different mechanisms including: ocular hypotony, local choroidal and scleral thickening, tangential vitreoretinal traction, inward folding of the sclera through collapsed posterior portion of the eye wall, asymmetry in staphyloma progression, or resistance to deformation of scleral staphyloma [34]. Visually threatening macular complications have been suggested to be more frequent in eyes with dome-shaped macula than eyes with low or no myopia, which include myopic CNV, macular holes (MHs), serous RD, foveal and extrafoveal schisis [35]. In the current study the prevalence of such complications in dome shaped macula, was as the following: 10 eyes with myopic CNV, 20 eyes with full thickness macular hole, foveoschisis in 26 eyes, and 2 eyes with shallow serous retinal detachment.

There was a statistically significant positive correlation between AL and dome shaped macula (r=0.475 and P=0.001).

## 6 LIMITATIONS

To this study includes: the study was concerned with the frequency of OCT findings in highly myopic eyes, without studying the course of some important changes (MTM, foveoschisis, traction), so further longitudinal studies are required to establish the findings that are definitely associated with progression. Visually threatening macular complications occurring in eyes with dome-shaped macula, should be studied in correlation with the height of dome shaped macula.

## CONCLUSION

In this study, we demonstrated that retinal abnormalities in the macular area can be a frequent finding in eyes with degenerative myopia. Epiretinal traction and CNV play a major role in the prognosis of these cases.

Optical coherence tomography can explain patient's visual complaints in highly myopic eyes and may even show abnormalities in asymptomatic cases. It can also detect some undetectable abnormalities on routine ophthalmoscope examinations in order to avoid advanced retinal damage.

## Figures and Tables

**Fig. (3):**
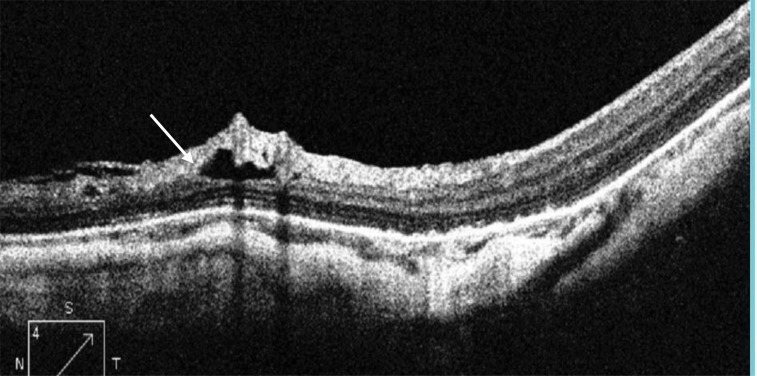
HD OCT scan showing paravascular retinal cyst (white arrow).

**Fig. (5):**
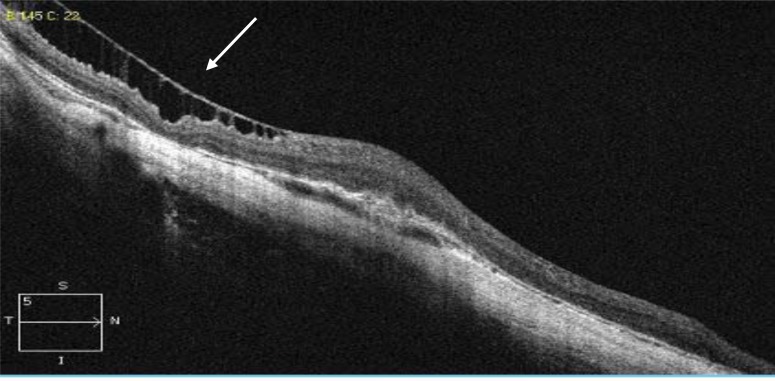
HD OCT scan showing tractional internal limiting membrane (ILM) detachment (white arrow).

**Fig. (9):**
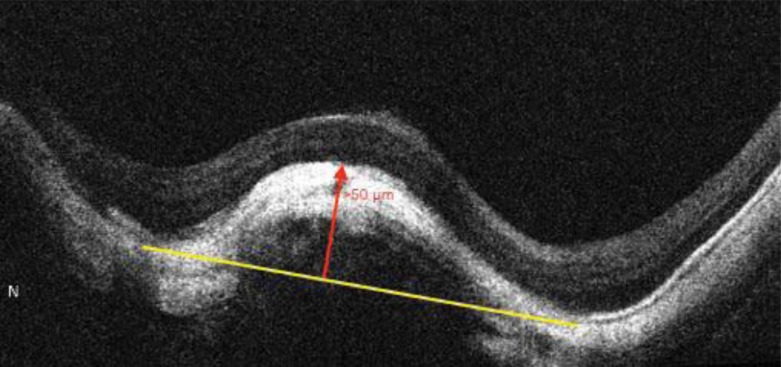
HD OCT scan showing a dome shaped macula, which is defined as inward bulge of RPE of more than 50 µm (red arrow), above a presumed tangential line (yellow line) joining the outer border of RPE, at the bottom of the posterior staphyloma.

**Table 1:** Patients' age, SE (spherical equivalent), and AL (axial length).

	Range	Mean ±SD
Age	22 to 65 years	50.38±11.4 years
Spherical equivalent	-6.50 to -23D	-13.11± 4.35D
Axial length	26 to 33.85 mm	28.5±1.62 mm

**Table 2:** The frequency of OCT findings.

	Number of cases	Percentage
Lamellar macular hole	22	4.6%
Full thickness macular hole	39	8.2%
Retinal detachment	18	3.8%
Inactive CNV	34	7.2%
Active CNV	85	18.0%
Myopic Foveoschisis	104	22%
Epiretinal membrane	74	15.6%
Myopic traction maculopathy	61	12.9%
Paravascular retinal cyst	33	6.9%
Vascular microfolds	10	2.1%
Tractional (ILM) detachment	50	10.5%
PVD without macular traction	93	19.7%
PVD with macular traction	65	13.7%
Dome shaped macula	58	12.2%

**Table 3:** The frequency of some OCT findings between eyes with AL >28mm, and eyes with AL >30 mm.

OCT findings	Axial length> 28mm	Axial length>30 mm
Lamellar macular hole	15 (3.11%)	12 (2.5%)
Full thickness macular hole	27 (5.7%)	23 (4.8%)
Retinal detachment	16 (3.3%)	14 (2.9%)
Active CNV	39 (8.2%)	32 (6.7%)
Foveoschesis	100 (21.1%)	93 (19.7%)
Myopic traction maculopathy	48 (10.1%)	45 (9.5%)
Dome shaped macula	57 (12%)	55 (11.6)
